# Cdk8 Kinase Module: A Mediator of Life and Death Decisions in Times of Stress

**DOI:** 10.3390/microorganisms9102152

**Published:** 2021-10-15

**Authors:** Brittany Friedson, Katrina F. Cooper

**Affiliations:** Department of Molecular Biology, Graduate School of Biomedical Sciences, Rowan University, Stratford, NJ 08084, USA; frieds15@rowan.edu

**Keywords:** cyclin C, Med13, cellular stress, regulated cell death, hyper-fission, autophagy, proteasome, mitochondrial dynamics, ROS and nutrient signaling

## Abstract

The Cdk8 kinase module (CKM) of the multi-subunit mediator complex plays an essential role in cell fate decisions in response to different environmental cues. In the budding yeast *S. cerevisiae*, the CKM consists of four conserved subunits (cyclin C and its cognate cyclin-dependent kinase Cdk8, Med13, and Med12) and predominantly negatively regulates a subset of stress responsive genes (SRG’s). Derepression of these SRG’s is accomplished by disassociating the CKM from the mediator, thus allowing RNA polymerase II-directed transcription. In response to cell death stimuli, cyclin C translocates to the mitochondria where it induces mitochondrial hyper-fission and promotes regulated cell death (RCD). The nuclear release of cyclin C requires Med13 destruction by the ubiquitin-proteasome system (UPS). In contrast, to protect the cell from RCD following SRG induction induced by nutrient deprivation, cyclin C is rapidly destroyed by the UPS before it reaches the cytoplasm. This enables a survival response by two mechanisms: increased ATP production by retaining reticular mitochondrial morphology and relieving CKM-mediated repression on autophagy genes. Intriguingly, nitrogen starvation also stimulates Med13 destruction but through a different mechanism. Rather than destruction via the UPS, Med13 proteolysis occurs in the vacuole (yeast lysosome) via a newly identified Snx4-assisted autophagy pathway. Taken together, these findings reveal that the CKM regulates cell fate decisions by both transcriptional and non-transcriptional mechanisms, placing it at a convergence point between cell death and cell survival pathways.

## 1. Introduction

Several age-related maladies, including cancers and neurodegenerative diseases, are associated with deteriorating cellular functions. This can result in the loss of cellular homeostasis and decreased ability to elicit correct cell fate responses to unfavorable environmental cues [1]. For instance, high reactive oxygen species (ROS) levels induce protein and organelle damage, stimulating regulated cell death (RCD) pathways in both yeast and mammals [2,3,4,5]. RCD induction involves transcriptional changes and disruption of mitochondrial integrity. In contrast, pro-survival responses are initiated by different cellular cues including nutrient depletion [6]. Under these conditions, the cell induces a different transcriptional response to help recycle cellular components to refill substrate pools and maintain mitochondrial integrity to maximize energy resources. Thus, successfully interpreting a specific stimulus requires the seamless integration of transcriptional remodeling with organelle function.

The molecular details of how cells execute cell death and cell survival pathways are reasonably well understood. Significantly less clear is how cells decide which path to follow in response to different stressors. Our work and those of others in budding yeast and mammalian cells alike have provided evidence that the Cdk8 kinase module (CKM) of the Mediator complex plays a critical role in these decisions [7,8,9,10,11,12,13,14,15,16,17]. The CKM is a dissociable part of the Mediator complex and contains four subunits- Med12, Med13, Cdk8, and cyclin C. In yeast, CKM association with the Mediator predominantly represses transcription [11,18,19], although positive roles have been described [20,21,22,23]. At the mechanistic level, how the CKM executes these different roles is unclear, but likely dependent on specific promoter contexts [24,25]. Deciphering the contribution of the CKM to diverse biological outputs is important as the dysfunction of any of its members is linked to a multitude of human disease, including cancer [26]. Moreover, in mammalian cells, the kinase module can also encompass the paralogs CDK19, MED12L, and MED13L [9]. Genetic variations in these paralogues are also linked to an overlapping spectrum of disorders [27]. Interestingly, no paralogue of cyclin C has been identified. Consistent with this, cyclin C forms a distinct Mediator kinase module with CDK19, which regulates a different transcriptional program to cyclin C-CDK8 [28,29]. In this review, we discuss these transcriptional roles of the CKM. Thereafter, we discuss the significance of cyclin C’s secondary cytoplasmic role as an envoy of nuclear decisions following cell death or survival cues.

## 2. The CKM Regulates Cell Fate Decisions

**Figure 1:**
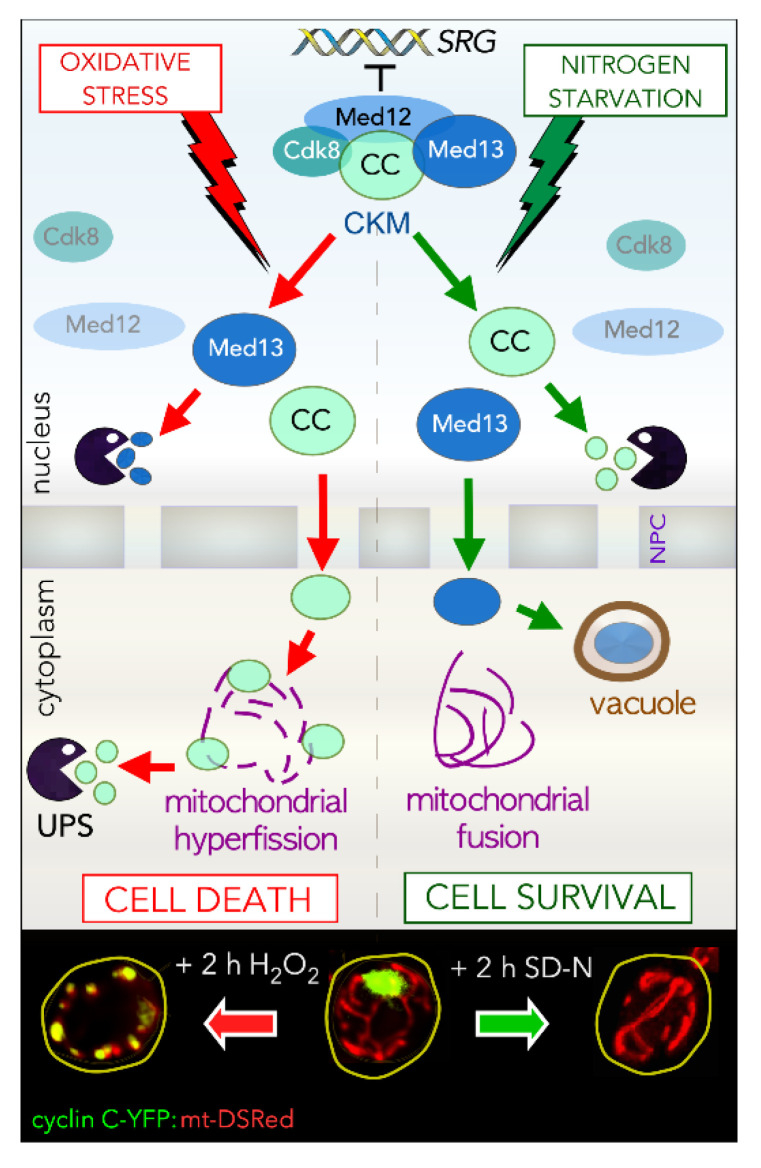
Top panel: Model outlining the different mechanisms used by the CKM to relieve repression on SRG’s in response to either cell death (oxidative stress) or cell survival (nitrogen starvation) cues in yeast. Bottom panel: Live fluorescence images of cyclin C-YFP and DS-Red tagged mitochondria demonstrating the different phenotypes observed after 2 h treatment with H_2_O_2_ (left) or following nitrogen starvation (SD-N—right). CKM-Cdk8 kinase module, SRG’s—stress response genes, YFP—yellow fluorescence protein.

In addition to its transcriptional role, the CKM regulates cell fate decisions by modulating the sub-cellular address of cyclin C (Figure 1). Several types of cellular damage induce cyclin C nuclear release and association with the mitochondria [30,31,32,33,34,35,36,37,38,39,40,41,42,43]. This association triggers extensive mitochondrial fission (hyper-fission) and promotes cell death initiation. Underlying the significance of both roles, these “day and night jobs” of cyclin C are conserved from yeast to mammalian cells, although there are some differences that are discussed below and summarized in Table 1 [13,41]. These studies are also consistent with the emerging theme that proteins can have two different functions, coined “day and night jobs”, which are summoned by different external or intrinsic stimuli [44,45]. Although the transcriptional response to nutritional deprivation overlaps with exposure to cytotoxic compounds, the fate of cyclin C is very different. Although still destroyed to allow SRG derepression following nitrogen starvation, cyclin C does not make it to the cytoplasm, as observed with oxidative stress. Rather, it is destroyed via the UPS prior to its nuclear release [37]. Why is this important? Under starvation conditions, the cell wants maximum ATP production, which requires reticular mitochondria. Inducing fragmentation would be counterproductive. Second, mitochondrial localization of cyclin C pushes the cell toward RCD [37]. This is best illustrated by ectopically targeting cyclin C to the mitochondria [41]. Although mitochondrial fission is induced, cell viability is normal. However, with cyclin C at the mitochondria, nitrogen starvation now triggers the cell death pathway, not a survival response [37]. These findings indicate that cellular outputs to a given signal can be switched simply by altering the location of a single protein. Below, we summarize the molecular details gleaned to date on how the CKM controls cell fate decisions, discussing the impact of cyclin C’s transcriptional and post-transcriptional roles (day and night jobs) on this process.

## 3. The Transcriptional Role of the CKM in Response to Stress

### 3.1. Association of the CKM with the Core Mediator

**Figure 2:**
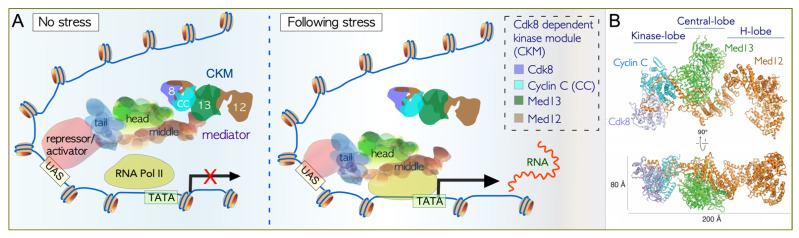
Model of the interaction of the CKM with the core mediator complex and RNA Pol II. (**A**). In unstressed cells, the CKM associates with the Mediator at UAS sites found in promotors, by the interaction of Med13 with the mediator hook. This inhibits the Mediator-RNA pol II interaction, preventing PIC formation and transcription of mRNA. Following stress, the CKM is released from the Mediator, permitting PIC assembly and transcription of mRNA. Additional repressors and/or activators also mediate transcription by binding to UAS motifs (Upstream Activating Sequence). Adapted from Cherji et al (2017). NAC. PMID:28575439. (**B**) Structure of the yeast CKM determined by cryo-EM and mass spectrometry. Reproduced with permission from Li et al (2021) Scientific Advances. PMID: 33390853. CKM—Cdk8 kinase module, RNA Pol II—RNA polymerase II, PIC—preinitiation complex, Å—Angstrom.

The core Mediator complex is a large multi-subunit coactivator complex organized into three functional modules coined Head, Middle, and Tail. Its modular architecture and subunit composition are conserved [46], which allows it to communicate signals originating from a plethora of transcription factors (TFs) to facilitate RNA pol II-directed transcription. This is aided by large intrinsically disordered domains (IDRs) found within its subunits, providing the Mediator with a high degree of structural flexibility [47,48,49]. A combination of in vitro and in vivo approaches (reviewed in [50]) has uncovered mechanistic details into how the CKM interacts with the Mediator (Figure 2A). One model for transcriptional repression proposes CKM binding to the Mediator precludes the interaction of the RNA pol II at gene promotors [9,51,52]. Recent structural studies have suggested that CKM mediated phosphorylation of other core Mediator subunits releases this steric inhibition [53]. CKM release from the Mediator allows pre-initiation complex (PIC) assembly and is a prerequisite for transcription initiation [7,52,54,55,56,57]. In mammalian systems, the CKM is recruited to promoters that are being actively transcribed [14,58]. Several mechanisms have been proposed for its positive role in transcription, including modifying other regulators [59,60]. These studies provide mechanistic insights into how CKM activity stimulates transcription of genes in stress and signal response networks [13,14,61]. Understanding CKM regulation is important, as its dysregulation is implicated in over 100 different human cancers [62,63,64]. 

In yeast, the CKM predominately negatively regulates a subset of SRG’s, including genes encoding antioxidants, chaperones and proteins needed for autophagy [8,18,30,33,65]. Consistent with this, CKM members were initially identified as suppressors of aberrantly expressed SRG’s in separate genetic screens [8,17,66,67]. Cyclin C and Cdk8 were also isolated in a screen looking for suppressors of truncation of the yeast C terminal tail of RNA pol II [68]. Unfortunately, this has led to significant nomenclature confusion, with CKM members having multiple gene names. To circumnavigate this, in 2004, the Mediator nomenclature unification effort [69] reclassified CKM members to the following names: cyclin C (*CNC1-* old names: *SSN8*, *UME3*, *SRB11*, *RYE2,* and *GIG3*), Cdk8 (*CDK8*- old names: *SSN3*, *UME5*, *SRB10*, *RYE5,* and *GIG2*), Med12 (*MED12-* old names: *SSN5*, *GIG1,* and *NUT6*) and Med13 (*MED13-* old names: *SSN2*, *UME2*, *SRB9*, *RYE3,* and *NUT8*).

### 3.2. Cdk8 Is a Non-Canonical Cyclin-Dependent Kinase

Cyclin-dependent kinases (CDKs) are serine/threonine protein kinases that fall broadly into two functional groups as regulators of cell cycle progression or transcription [70]. CDK activation requires cyclin binding with the interaction stabilized by CDK activating kinase (CAK) phosphorylation in the CDK T-loop [71]. However, CDK8 is distinct among the CDK family as it lacks a canonical phosphorylation residue within its T-loop [72]. Instead, biochemical experiments suggest that human MED12 is essential for stabilizing the CDK8–cyclin C association within the module [73]. Importantly, known oncogenic *MED12* mutations [74,75] disrupt the ability of MED12 to activate, but not bind, cyclin C-CDK8. These results are consistent with the model that MED12 binding is necessary, but not sufficient, for efficient CDK8 activation. Recent structural studies of the yeast CKM have provided significant insights into this proposed model, revealing that Med12 stabilizes Cdk8 in an activated conformation (reproduced with permission in Figure 2B). This triggers a structural rearrangement that contributes to the stabilization of the Cdk8 T-loop, thereby enabling kinase activity in the absence of canonical T-loop phosphorylation [76]. In the absence of Med12, the Cdk8 T-loop is flexible, and unfavorably poised for substrate binding and phosphorylation. Consistent with this, both Med13 and Med12 are essential for the transcription of Wingless target genes in *Drosophila* [77].

### 3.3. Dissociation of the CKM from the Core Mediator

The mammalian CKM both negatively and positively regulates SRG’s in approximately equal numbers [13,78]. Our recent studies of promoter occupancy in mammalian cells revealed that CKM dissociation from promoter regions coincides with derepression of negatively regulated mRNAs [14]. These studies, as well as those by others, showed that chemical inhibition of CDK8 and CDK19 kinases removes their ability to repress the Mediator complex at enhancers [79]. Conversely, genes that require the CKM for transcription induction exhibited increased promoter occupancy. The molecular mechanisms underlying the reversible CKM-Mediator association are incompletely understood but center on Med13, the largest subunit in Mediator, which anchors the CKM to the hook domain formed by Middle module subunits [7,55,80]. Studies in both humans and yeast indicate that Med13 phosphorylation, ubiquitylation, and turnover all control CKM-Mediator association [36,40,81]. Very recent cryo-EM and mass spectrometry (XL-MS) studies [76] have provided a 3D structural representation of the yeast CKM, detailing the interaction of multiple lobes (Figure 2B). The kinase-lobe (composed of cyclin C and Cdk8) and the H-lobe (composed of Med12) protrude from a central-lobe containing Med13 and Med12. Consistent with previous studies, cyclin C joins Cdk8 in the kinase lobe and interacts with the scaffold Med12 [7,76,82]. These findings significantly improve our understanding of how the CKM regulates transcription, providing insights into why the CKM is implicated in a growing number of cancers and developmental diseases [29,64,79,83,84]. Unexpectedly, they also reveal that yeast Med13 has an Argonaute-like bi-lobal architecture, although the functional significance of this finding remains unknown [76].

### 3.4. Disassembly of Cyclin C from Med13

As the translocation of cyclin C into the cytoplasm following oxidative stress sets cells on a path to RCD, it is vital to understand how cyclin C is released from its CKM anchor, Med13. Genetic manipulations of cyclin C localization in yeast revealed that cyclin C contains a conserved holoenzyme association domain (HAD) located at the N terminus of cyclin C. This domain consists of a short alpha-helical region containing a conserved “KERQK” sequence required for Med13 binding [85]. HAD domain deletion causes cyclin C dissociation from the CKM and reduction of transcriptional repressor ability. Moreover, similar to the *med13*∆ phenotype, cyclin C*^HAD^*^∆^ mutants colocalize with mitochondria causing fragmentation in unstressed cultures [40]. Taken together, this supports the model that cyclin C disassembly from the CKM plays a role in its localization to the mitochondria [34]. To demonstrate that the cyclin C HAD-MED13 interaction is conserved, Jezek et al. [38] designed a cell-penetrating peptide mimetic (S-HAD) that effectively disrupts Med13-cyclin C interaction. When S-HAD was added to unstressed mouse embryonic fibroblasts (MEFs), it rapidly induced cyclin C nuclear release and mitochondrial fragmentation. The S-HAD-induced mitochondrial fragmentation is dependent on cyclin C, as this response was not observed in *Ccnc* null (*Ccnc*^−/−^) MEFs [38]. These findings indicate that the interaction between Med13 and cyclin C is conserved, and that cyclin C nuclear release is sufficient to induce mitochondrial fragmentation in the absence of additional stress signals.

### 3.5. Genes Regulated by the CKM in Response to Cell Death Cues

The transcriptome is significantly remodeled to adapt to unfavorable environmental cues [86,87,88,89]. This requires the activation of transcription factors, many of which are conserved, including CKM, FOXO, HSF1, and NRF2 [88,90,91,92,93,94]. For example, oxidative stress stimulates transcription factor activity upregulating many genes encoding antioxidants (catalases) and pro-survival chaperones [95] required to reinstate cellular homeostasis. Failure to neutralize the toxic effects of reactive oxygen triggers cells to switch to upregulating genes required for cell death pathways [2,96]. Mechanistic details on how cells make this molecular switch remain unclear, but the CKM plays a key role. Relief of repression by the CKM is mediated by CKM removal from the core Mediator. Furthermore, quantitative PCR analysis revealed that cyclin C nuclear release in yeast following oxidative stress is required for full mRNA accumulation of *DDR2* and *CTT1* [32], genes that encode a multi-stress response protein and catalase, respectively. Moreover, a cis-acting cyclin C mutant (A110V) remained nuclear following stress while preserving its normal transcriptional role [32,33]. Lastly, a significant reduction in viability was observed in H_2_O_2_-treated cells harboring the A110V mutant as compared with wild type, implicating a role for the cyclin C promoter removal to properly regulate RCD [31]. 

### 3.6. Genes Regulated by the CKM in Response to Cell Survival Cues

In response to nitrogen starvation, autophagy (*ATG*) genes are upregulated to promote survival [97]. This is mediated by various transcriptional regulators including the CKM [37,98,99]. Epistasis analysis revealed that CKM regulation of *ATG8*, which is required for phagosome formation, occurs within the Rpd3–Sin3–Ume6 histone deacetylase (HDAC) axis [37,100]. The molecular mechanisms controlling this derepression remain unclear. However, the events downstream of CKM dissociation are different from those occurring in oxidative stress. Significantly, following nitrogen starvation, the UPS destroys cyclin C before its detection in the cytoplasm [37,98,101]. The destruction of cyclin C thus contributes to the expression of genes involved in yeast autophagy. While CKM deletion in the absence of stress is not sufficient to induce autophagy, yeast cells lacking cyclin C-Cdk8 demonstrate a growth advantage due to increased *ATG8* induction. Likewise, the presence of cyclin C-Cdk8 restricts the growth of cells partially inhibited for TORC1 [37]. These data suggest that loss of cyclin C-Cdk8 following nitrogen starvation stress enhances cell survival by relieving repression of SRG, though more studies are needed to flush out molecular details. The degradation of cyclin C-Cdk8 can thus adjust a cell’s pro-death or pro-survival transcriptional program depending on the environmental trigger. Similarly, the CKM controls cell fate decisions during development of B and T cells as well as during hematopoiesis [102,103].

## 4. Upstream Signaling Pathways Link Degradation Machines to Stress

### 4.1. Oxidative Stress Activates the Cell Wall Integrity Pathway

**Figure 3:**
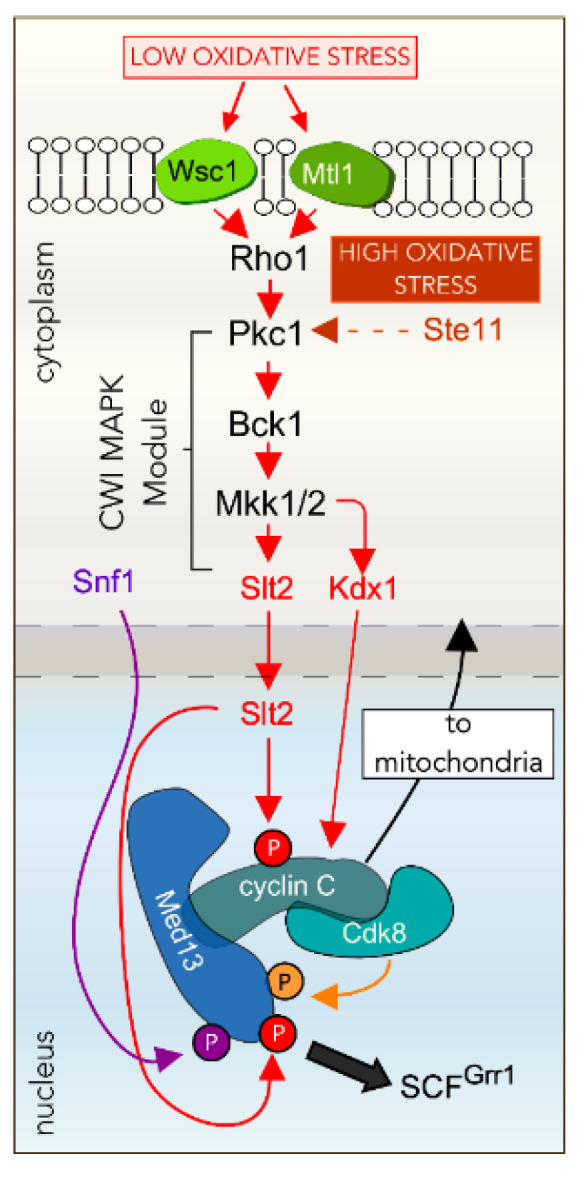
Outline of the Cell Wall Integrity (CWI) signal transduction pathway that mediates cyclin C translocation to the cytoplasm following low oxidative stress (0.4 mM H_2_O_2_). Both the MAPK of this pathway (Slt2) and its pseudo-kinase partner, Kdx1 are required for the efficient export and degradation of cyclin C. Cyclin C release is dependent upon Med13 degradation by the SCF^Grr1^ E3 ligase complex, triggered by Cdk8, Slt2 and the AMPK Snf1-mediated phosphorylation. Following high levels of oxidative stress (1.2 mM H_2_O_2_) the activity of Ste11, the MAPKKK of the HOG signal transduction pathway is also required. For clarity, Ask10 and Plc1, which also play roles in cyclin C’s fate are omitted from the figure. MAPK—mitogen activated protein kinase, SCF—Skp1-Cullin-F-box-protein, HOG—high osmolarity glycerol, AMPK—adenosine monophosphate-activated protein kinase, P—phosphorylation event.

The Cell Wall Integrity (CWI) pathway is the main signaling pathway involved in the regulation of cell wall stress responses [104,105]. A family of cell-surface sensors (Wsc1, Mid2, and Mtl1) communicate the environmental stress signal to a small G protein Rho1, which further activates protein kinase C (Pkc1) [89,106]. Activated Pkc1 triggers the linear mitogen-activated protein kinase (MAPK) cascade (Figure 3). This is comprised of the MAPKKK kinase (Bck1), a pair of redundant MAPKK’s (Mkk1/2), a MAPK (Slt2/Mpk1), and a pseudo-kinase (Ksp1/Mlp1) [89]. Slt2 is a functional homolog of human extracellular signal-regulated kinase 5 (ERK5) that is also activated in response to physical and chemical stresses [107]. Phosphorylation of Slt2 directly activates two transcription factors: Rlm1, which induces transcription of a wide array of cell wall metabolism genes [108], and SBF, a dimeric transcriptional regulator comprised of Swi4 and Swi6, required for upregulation of G1-specific genes [109]. 

### 4.2. The CWI MAPK Slt2 Is Required for Cyclin C Nuclear Release in ROS

Slt2 directly phosphorylates both cyclin C and Med13 in response to oxidative stress (Figure 3) [35,36]. The phosphorylation of cyclin C on serine 266 is required for its nuclear release [35], whereas the phosphorylation of Med13 on threonine 835 and 837 triggers its destruction by the multi-subunit E3 ligase SCF^Grr1^. Genetic analysis also revealed that Med13 destruction requires a priming event mediated by Cdk8 in unstressed cells [36]. Thus, typical of SCF E3 ligases, recognition of the Med13 degron uses two phosphorylation marks, one to prime the degron, and the second for its recognition by ubiquitin ligases [110,111]. Additionally, Med13 destruction requires the direct phosphorylation by Snf1 on a residue adjacent to the Slt2 sites in Med13’s IDR domain [112]. Snf1 is a highly conserved adenosine monophosphate-activated protein kinase (AMPK) that is activated in response to a variety of stresses, including oxidative stress [112,113]. As multiple signaling pathways converge on the CKM, this reveals that cells place several checks on this system. This ensures that releasing cyclin C to the cytoplasm is the correct response to the environmental input.

### 4.3. Other Factors Required for ROS-Dependent Cyclin C Nuclear Release

The nuclear release of cyclin C is also known to require the Slt2 paralogue Kdx1, the transcription factor Ask10, and the conserved signaling enzyme phospholipase C (Plc1) [33,35,114]. Mechanistically, it remains unknown how these factors contribute to cyclin C nuclear release. This further emphasizes that cyclin C nuclear release is highly regulated.

### 4.4. Cyclin C Nuclear Release Requires Additional MAPK Pathways in Response to More Stringent ROS

In response to mild oxidative stress (induced by 0.4 mM H_2_O_2_), reprograming a single MAPK pathway is sufficient to induce the yeast cyclin C nuclear translocation and eventual destruction. However, increasing the severity of this environmental challenge (1.2 mM H_2_O_2_) triggers a more extensive cellular response mediated by cross-pathway signaling. This is accomplished by recruiting Ste11, a MAPKKK traditionally used by the high osmolarity glycerol (HOG) and mating-type signaling pathways [115]. However, under these severe conditions, Ste11 activates Bck1, the MAPKKK of the CWI pathway [116,117]. The HOG and CWI pathways traditionally were thought to be separate [118,119], but now, similar to other signal transduction pathways, exhibit considerable crosstalk in response to different signals [120]. This ability to discriminate stress intensity by remodeling signaling pathway architecture provides the cell a mechanism to speed up the timing of the response as well as providing backup strategies to ensure that the stress signal is effectively transmitted to the nucleus.

### 4.5. Nitrogen Starvation Inhibits TORC1

The conserved target of rapamycin kinase complex 1 (TORC1) signaling pathway couples environmental and nutritional cues to downstream effectors. TORC1 phosphorylates a wide range of targets that drive protein, lipid, and nucleotide synthesis [121] and is often dysregulated in human diseases, including cancer, type 2 diabetes, and neurodegeneration [122,123]. Its core, ring-like structure is highly conserved, consisting of a heterotrimeric complex that harbors a TOR serine/threonine protein kinase (TOR1 or TOR2 in yeast) and two regulatory proteins called Kog1 and Lst8 [124]. A fourth protein called Tco89 associates with this core complex to adapt its function to species-specific requirements [125]. 

Nitrogen and amino acid signals are transmitted to TORC1 by the heterodimeric Rag guanosine triphosphatases (GTPases). In yeast, these are encoded by Gtr1 and Gtr2 [126]. In the presence of abundant nitrogen/amino acids, Gtr1 and Gtr2 are in their GTP, and GDP bound forms, respectively, and bind tightly to Kog1 and Tco89 [127]. In contrast, when nutrient or energy levels fall, TORC1 is inhibited, causing cells to switch from anabolic to catabolic metabolism, eventually entering quiescence [128]. The binding of another complex called SEACIT to Gtr1 induces its GTPase activity [129]. The resulting GDP-bound Gtr1 consequently causes a conformational change that weakens the interaction between Gtr1/2 and TORC1 and rapidly inhibits TORC1 signaling [130].

In yeast, Rag GTPase is tethered to the vacuole membrane by the EGO complex (EGOC), resulting in constitutive localization of TORC1 to this organelle [127,131]. Additionally, a newly identified second pool of TORC1 that localizes to the pre-vacuolar endosome [132] has led to an updated model by which these spatially separated pools of TORC1 execute distinct functions [133]. Once activated, the vacuolar pool of TORC1 primarily upregulates protein translation by phosphorylating the AGC-kinase family protein Sch9 [134]. In contrast, the endosomal pool predominantly inhibits autophagy by phosphorylating Atg13 and Vps27 respectively [132,135].

### 4.6. Nitrogen Starvation Activated Slt2 Does Not Directly Phosphorylate Cyclin C

It is unclear how the stress signals are transmitted to cyclin C following TORC1 inhibition. Similar to ROS stress, Slt2 is activated under these conditions [136], but how TORC1 communicates with the CWI MAPK pathway is unclear. Other roles of activated Slt2 under nitrogen depletion include directing G1 arrest and entrance into quiescence. Here, in a series of elegant experiments, Moreno-Torres et al. [137,138] discovered that Slt2 mediates the phosphorylation of the cell cycle-dependent kinase inhibitor (CDKI) Sic1. This targets Thr173, a different phosphorylation signature from those that trigger Sic1 destruction, converting this CDKI into a potent inhibitor of Clb5-CDK-Cks1 complexes [137,138]. Intriguingly, the only MAPK site in cyclin C (Ser266) is not required for its proteolysis following TORC1 inhibition [37]. These findings suggest that Slt2 functions indirectly to target cyclin C for destruction. Alternatively, as Slt2 can also target tyrosine residues [139,140], this mechanism could be used to transmit the signal to cyclin. Nevertheless, these results are consistent with the model that the differential regulation of cyclin C contributes to its final subcellular address, which consequently contributes to cell fate decisions. 

### 4.7. Cyclin C Destruction in Nitrogen Starvation Is Mediated by the UPS

A recent study in our laboratory uncovered that yeast cyclin C is degraded following nitrogen starvation or rapamycin treatment by the UPS before its detection in the cytoplasm [37]. Similar to ROS stress, cyclin C degradation requires both the 20S catalytic core particle and the 19S regulatory particle of the 26S proteasome. Interestingly, cyclin C is ubiquitinated using the same E2 ubiquitin conjugating enzymes, Ubc4 and Ubc5, following either stress [32,37]. In contrast, the E3 ubiquitin-ligating enzyme Not4, which mediates cyclin C degradation following oxidative stress, is not required for its degradation in nitrogen starvation. Moreover, similar to other substrates [141,142,143,144,145], multiple Ub ligases mediate cyclin C proteolysis during nitrogen starvation, as no single E3 ligase fulfils this role [37]. The necessity for the same E2 enzymes, but a different E3 ligase following each stress, emphasizes the precision in which ubiquitin can tag cyclin C to be recognized by the proteasome at a specific time and cellular location. Uncovering the E3’s that mark cyclin C for degradation following nitrogen starvation will provide insight into the mechanisms used to differentially regulate cyclin C and how this contributes to cell survival.

### 4.8. TORC1 Inhibition Results in Med13 Degradation by Snx4-Assisted Autophagy

**Figure 4:**
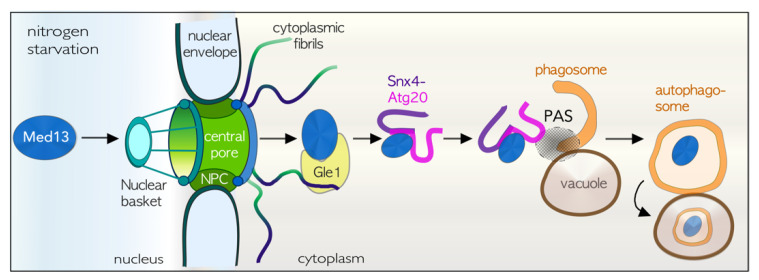
Upper panel: Outline of the Snx4-assisted autophagy pathway of transcription factors. This pathway transports Med13 from the nucleus to the vacuole for proteolysis following TORC1 inhibition. NPC—nuclear pore complex, PAS—Pre-autophagosomal structure.

As cyclin C nuclear release requires Med13 destruction by the SCF following cell death cues (particularly ROS), we were surprised to find that cells use a different mechanism to destroy Med13 after nitrogen depletion. In these conditions, Med13 degradation occurs by vacuolar proteolysis requiring the autophagy machinery [42]. In this pathway, outlined in Figure 4, Med13 shuttles through the nuclear pore complex (NPC), associating first with the cytoplasmic nucleoporin Gle1, a member of the RNA remodeling complex [146,147,148,149,150]. Next, Med13 is transported to Atg17-initiated phagophores located on the vacuole [151,152], aided by the sorting nexin heterodimer Snx4-Atg20. Lastly, upon fusion of the autophagosome with the vacuole, Med13 proteolysis occurs with Snx4-Atg20, being recycled back to the cytosol. In addition, the degradation of Rim15 and Msn2, transcriptional activators of *ATG* genes, also occurs by this pathway [42]. As turnover of both positive and negative regulators of *ATG* transcription is controlled by Snx4-assisted autophagy (SAA), this pathway permits cells to fine-tune the autophagic response. Moreover, the pathway is distinct from known nucleophagy mechanisms [153,154], which remove unspecified nuclear material through blebbing of the nucleus [155].

### 4.9. The Role of Snx4 in Autophagy

The evolutionarily conserved sorting nexin Snx4 [156,157] is associated with the etiology of neurodegenerative diseases and cancer [158,159]. Despite this, its precise roles in autophagy and stress-induced cell survival remains elusive. Degrading transcription factors had not been a function previously assigned to Snx4-Atg20. This sub-group of sorting nexins all contain a characteristic BAR (Bin/|Amphiphysin/Rvs) domain that binds curved membranes upon dimerization [160,161]. The BAR domain also binds and transports a variety of cargos, including Med13 [42,162]. Snx4-Atg20 is also required for other selective autophagy pathways, including proteophagy, ribophagy, and a recently identified pathway used to degrade specific translating mRNA’s [163,164]. Underlining its importance, Snx4-Atg20 transports cargos from the vacuole to the Golgi, an essential function for autophagy [165,166]. Despite these varied roles, how Snx4-Atg20 recognizes its autophagic cargos is a topic for further investigations.

## 5. The Relationship between Mitochondria, the CKM, and Cell Fate Decisions

### 5.1. Control of Mitochondrial Dynamics

Mitochondria are not only sites of ATP generation by oxidative phosphorylation (OXPHOS), but also have additional roles, including calcium homeostasis and RCD regulation. In yeast and mammals, mitochondria form dynamic tubular networks constantly undergoing fission and fusion events. The equilibrium between fission and fusion is controlled by the activity of conserved molecular machines driven by dynamin-like GTPases [167]. Dysfunctional mitochondrial dynamics is associated with a broad range of human diseases, from cancer to neurodegenerative disorders [168]. Mitochondrial fission in mammalian cells is controlled by the highly conserved dynamin-like GTPase called DRP1 [169]. DRP1 is recruited to the outer mitochondrial membrane (OMM) by several adapter proteins, including mitochondrial fission factor (MFF), mitochondrial dynamics protein 49 and 51 (MiD49, MiD51), and mitochondrial fission 1 protein (hFis1) [170]. DRP1 self-assembles into filaments that form rings around the mitochondria. These rings constrict following GTP hydrolysis with the final scission being executed by dynamin 2 (DNM2) [171,172,173]. This concept is conserved in yeast, where a single OMM protein, Fis1, interacts with one of two adaptor molecules (Mdv1 or Caf4) recruiting the GTPase Dnm1 to mitochondria [174,175,176,177,178,179]. Mitochondrial fusion in yeast is initiated through the Fzo1 (outer membrane, OMM) and Mgm1 (inner membrane, IMM) GTPases [180,181]. 

### 5.2. Mitochondrial Dynamics in Unstressed Cells

**Figure 5:**
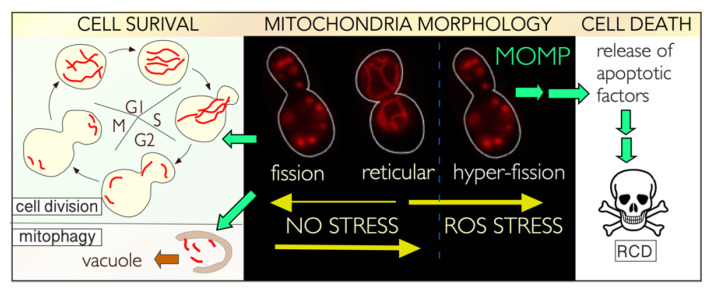
Outline of the different mitochondrial dynamics observed in unstressed and ROS (Reactive oxygen species)-treated yeast cells. See text for details. The fluorescence images are of budding yeast harboring a Mt-DSRed plasmid that localizes to the OMM (outer mitochondrial membrane). MOMP—mitochondria outer membrane permeabilization, RCD—regulated cell death.

The balance between fragmented and fused mitochondria in unstressed cells is both asynchronous and adapted according to need [182,183,184]. In unstressed cells, mitochondria predominantly maintain reticular morphology, allowing maximum ATP production as well as membrane and mtDNA damage repair [185,186]. Mitochondria fission in unstressed cells (Figure 5) permits equitable inheritance of mitochondria during cell division [187,188]. In addition, fission is associated with the quality control process of mitophagy that degrades damaged mitochondria [189,190]. In mammalian cells, mitochondrial fission is a prerequisite for mitophagy, whereas in yeast, it enhances the process [189,191,192]. As dysfunctional mitochondria can leak significant levels of ROS from the electron transport chain, mitophagy plays a pivotal role in maintaining cellular homeostasis both during normal and stress conditions [193]. Failure to execute these functions is linked to the pathogenesis of many diseases, including Parkinson’s and Alzheimer’s [194]. 

### 5.3. Stress-Induced Mitochondrial Fission Is Linked to Cell Death Pathways

Extensive fission and loss of mitochondrial integrity is associated with RCD induction [195]. In mammals, the pro-death molecules BAX or BAK, which disrupts the OMM, are found at fission sites containing DRP1 and the mitofusin MFN2 [196,197,198,199]. The resulting BAX oligomer formation and MOM permeabilization (MOMP) [200] facilitate the release of pro-apoptotic factors, including cytochrome c and apoptosis-inducing factor (AIF), which initiate caspase-dependent or caspase-independent cell death [201,202,203]. Consistent with this model, DRP1 knockdown cells are resistant to RCD [204]. However, others report that fission itself is not necessary for the timely release of some pro-apoptotic proteins [205,206]. Therefore, the connection between the fission machinery and RCD remains to be clarified. As DRP1 is required for fission during both mitosis and RCD induction, the cell must distinguish these activities to correctly respond to either cell growth or cell death stimuli [184].

In yeast, many regulated cell death hallmarks are observed following stress treatment (e.g., H_2_O_2_ or acetic acid) [207,208,209]. A critical difference from mammalian RCD pathways is that yeast lack orthologues of BAX and BAK. However, many executioners of RCD are conserved, including cytochrome c, the nucleases Aif1 and Nuc1 (EndoG), and the metacaspase Mca1/Yca1 [210], although its exact role is still unclear. Stress-induced mitochondrial hyper-fission is also associated with the release of these sequestered apoptotic factors and *DNM1* deletion protects yeast cells from RCD [204,211]. As the release of pro-apoptotic factors from the mitochondria is a hallmark of RCD, it is generally accepted that yeast can execute RCD pathways following stress [209]. It also suggests an evolutionary linkage of these regulatory pathways [208].

### 5.4. Night Job of Cyclin C in Cell Death: A Mitochondrial Response

In addition to its transcriptional role, yeast cyclin C performs its stress-activated night job in the cytoplasm. Following H_2_O_2_ stress, cyclin C exits the nucleus and binds to the mitochondrial fission machinery, independent of Cdk8 (see Figure 1) [34]. Here it interacts physically and genetically with Mdv1 [34], stimulating Dnm1 GTPase activity [34]. Stress-induced cyclin C nuclear release is dependent upon Med13 destruction by the the SCF^Grr1^ E3 ligase complex and the UPS [36,40]. Interestingly, only cyclin C is released into the cytoplasm, leaving Cdk8 in the nucleolus [32]. After cyclin C has stimulated hyper-fission, the E3 ligase Not4 targets it for degradation by the UPS [30,32]. Genetic manipulations of cyclin C localization in yeast have supported this model. Here, constitutively placing cyclin C at the mitochondria by either deleting *MED13* [40] or by fusing cyclin C to the OMM binding region of Fis1 [37] results in hyper-fission in the absence of stress. Mutants that retain cyclin C in the nucleus (A110V, S266A) also result in significant reduction in mitochondrial fission following ROS stress as compared with wild-type cells [34]. These studies demonstrate that cyclin C localization to the mitochondria is necessary and sufficient for stress-induced mitochondrial hyper-fission. 

### 5.5. Conservation of the Night Job of Cyclin C

The mitochondrial role of cyclin C is highly conserved. In H_2_O_2_ stressed mammalian cells, a portion of nuclear cyclin C translocates to the mitochondria where it directly binds to DRP1, increasing its affinity to GTP and stimulating GTPase activity in vitro [41,43]. Consistent with the yeast results, adding recombinant cyclin C to permeabilized *Ccnc^−/−^* MEF cells induces complete fission [41]. Cyclin C also plays a role in the efficient mitochondrial localization and activation of BAX [38]. However, even though mitochondrial-associated cyclin C can recruit BAX to the OMM in the absence of stress, these cells do not undergo cell death as BAX fails to oligomerize. These data support a model in which cyclin C association defines an initial step in BAX-OMM recruitment and provides a physical connection between fission and apoptotic factors. The presence of cyclin C at the mitochondria allows the cell to discriminate stress-induced fission from other types of mitochondrial divisions. Consistent with this activity, cyclin C has been identified as a tumor suppressor in both Acute Lymphoblastic Leukemia [78] and thyroid tumors [64]. Moreover, the *CCNC* locus (6q21) [212] exhibits loss of heterozygosity in several cancers, including osteosarcoma [213]. CDK8 also has been implicated as an oncogene in an array of cancers including colon, breast, and prostate [214,215,216]. The roles of CDK8 defined in earlier studies [29,217,218] have provided the underlying framework for a partial understanding of its role in tumorigenesis.

### 5.6. Mitochondrial Dynamics in Nutrient Starved Cells

Much less is known about mitochondrial dynamics in response to amino acid or nitrogen starvation. In mammalian cells, nutrient starvation induces a hyper-fused mitochondrial network, capable of increased ATP production that promotes cell survival [192,219,220,221]. Mitochondrial fusion is achieved in part due to PKA-dependent phosphorylation of DRP1, which blocks OMM recruitment [219,220,222]. Elongated mitochondria are also observed in other conditions associated with increased ATP production [223]. Similarly, yeast grown with a nonfermentable carbon source require increased OXPHOS activity that is accompanied by elongated mitochondrial networks [224]. This is also partially mediated through PKA activity [225] and supports the model that high OXPHOS activity correlates with extensive mitochondrial networks [226].

### 5.7. A Different Night Shift for Cyclin C in Cell Survival: Preserving Mitochondrial Integrity

TORC1 inhibition also triggers cyclin C destruction, but does so prior to its detection in the cytoplasm [37]. This destruction enhances cell survival by allowing full derepression of SRG’s required for autophagy and other processes. In addition, this nuclear destruction precludes cyclin C relocalization to the cytoplasm, thus preventing mitochondrial hyper-fission. The importance of this switch in destruction strategy was tested by fusing cyclin C to an OMM protein in yeast. This fusion protein localizes to mitochondria and can induce mitochondrial fission without additional stress signals. Interestingly, subjecting these cells to nitrogen starvation initiated the cell death pathway, not the autophagic survival program [37]. This strongly suggests that cyclin C’s subcellular address is intricately linked to cell fates. Thus, these data imply that the nuclear degradation of cyclin C following TORC1 inhibition promotes survival by preventing cyclin C mitochondrial association. These findings argue that the systems controlling cell death and cell survival pathways are intimately involved. Interestingly, cell survival and cell death signals also induce different degradative fates for Med13 (Figure 1), being degraded by the UPS and SAA respectively [42]. These findings suggest that two CKM members have secondary cytoplasmic roles to play following different environmental cues. Moreover, our studies have shown that the correct execution of these roles fortifies the necessary molecular response to the incoming stress [31,34,37].

## 6. Conclusions: The CKM Is at the Crossroads of Different Cell Fates

Analysis of the CKM following stress has revealed that its role in mediating cell fate decisions extends beyond solely regulating SRG transcription. Relocalization of cyclin C to the mitochondria only in response to cell death cues suggests that the CKM is acting as a signaling system, transmiting decisions made in the nucleus to the organelles. This role is amplified during development, where reduced Cdk8 activity is linked to lower levels of cyclin C and could dictate early mammalian embryo decisions [79]. Thus, in stress and developmental scenarios, cyclin C is the CKM’s envoy, linking nuclear decisions to cellular outcomes in response to cell death cues.

**Figure 6:**
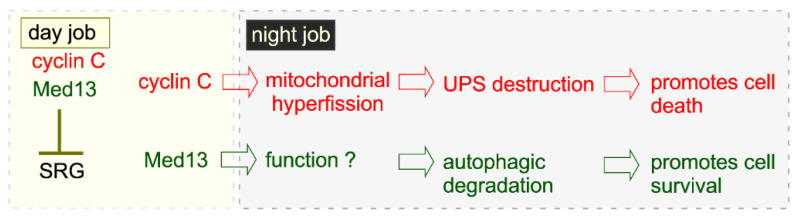
Similarities and differences between cyclin C and Med13’s state following stress. See text for details. SRG—stress response genes, UPS—ubiquitin proteasome system.

This model has led us to hypothesize that a similar signaling role may be assigned to Med13 in response to cell survival cues (Figure 6). The idea is supported by the observation that Med13 contains an exceedingly large (~700 kDa), intrinsically disordered region (IDR). IDR’s serve as flexible platforms for protein–protein interactions, which allow them to have many binding partners [227]. As such, IDR containing proteins often function as hubs for protein interaction networks, playing crucial roles in many biological processes including signaling pathways, transcriptional regulation, translation, and cell cycle control [228,229]. It comes as no surprise then that studies in Drosophila, Zebrafish, Arabidopsis, and mice have revealed that Med13 plays critical roles in embryonic and tumor development [230,231,232,233,234,235]. Moreover, germline variants of MED12 and MED12L also contain large IDR domains, and are found in several genetic disorders associated with X-linked intellectual disability, neuronal and developmental disorders, failure of hematopoietic-specific transcriptional programs, and cancer [84,102,236,237]. Intriguingly, many mutations in MED13L are found in its IDR [238] and result in a haplo-insufficiency syndrome characterized by a spectrum of symptoms including congenital heart and intellectual disorders [27,238,239]. Additionally, in yeast, the IDR associates with many proteins involved with Snx4-assisted autophagy [42]. Taken together, these findings support a model that this region of Med13 acts as an intricate communication hub for signaling proteins, thus allowing the proper cell fate response to environmental stress.

As highlighted in this review, *S. cerevisiae* is an excellent experimental model for understanding both cell death and cell survival responses with respect to CKM function [95,207,240]. Given the highly conserved nature of many players (see Table 1), its rapid growth rate, and facile gene manipulations, yeast will continue to provide critical insights into the molecular events that dictate life and death decisions. Importantly, this model system has been key to understanding the molecular events associated with selective and non-selective autophagy mechanisms, with 44 *ATG* genes known to date, most of which are highly conserved with mammalian autophagy pathways [77]. An immerging area for understanding cell fate decisions is the crosstalk between cell death and cell survival pathways. Rather than separate entities, these pathways share common regulators [4] whose relationships may be better dissected in a more tractable system such as yeast.

**Table 1:** Summary of the known roles of CKM following ROS stress and TORC1 inhibition in yeast and mammals.

Function	*S. cerevisiae*	Mammals
Predominantly negatively regulates SRG’s. Positively regulates a few genes.	[18,20,23,68,241,242]	No
Equally regulates positive and negative SRGs.	No	[13,14,28,243]
Cyclin C -Cdk8 phosphorylate the C-terminal domain of the largest subunit of RNA pol II in vitro, to inhibit transcriptional initiation.	[68,85,242]	[244]
Cyclin C -Cdk8 directly phosphorylates other Mediator subunit to negatively regulate transcription.	[245]	[246]
Cyclin C -Cdk8 phosphorylates transcription factors and other targets.	[247,248]	[241,246,249,250]
MAPK mediated phosphorylation of Med13 mediates its nuclear release following cell death cues.	[35]	unknown
Nuclear release of cyclin C requires Med13 degradation by UPS following cell death cues.	[36,40]	unknown
Med13 is regulated by SCF^Grr1/Fbw7^.	[36]	[81]
Cytoplasmic cyclin C mediates mitochondrial hyper-fission by binding to mitochondrial fission complex after ROS stress.	[34]	[41]
Cytoplasmic cyclin C promotes oligomerization of the GTPase DNM1/Drp1.	unknown	[43]
Cyclin C nuclear release is required for RCD (intrinsic pathway).	[31,34]	[41]
Cyclin C binds to BAX at mitochondria to induce MOMP following ROS stress.	No BAX	[38]
Cyclin C null mutants are deficient in stress-induced mitochondrial fission.	[34]	[41]
Cyclin C nuclear release after ROS stress promotes the release of pro-apoptotic factors.	Assumed [34]	[38]
Cyclin C is destroyed by the UPS in the cytoplasm in ROS stress after mediating mitochondrial hyper-fission.	[30,33]	No
ROS induced night job of cyclin C is independent of Cdk8.	[32]	[41]
CKM negatively regulates a subset of *ATG* genes.	[37,42]	[13,14]
Cyclin C is destroyed by the UPS following TORC1 inhibition	[37]	unknown
Destruction of cyclin C following TORC1 inhibition promotes cell survival.	[37]	unknown
Destruction of cyclin C following TORC1 inhibition promotes mitochondrial fusion.	[37]	unknown
Med13 is destroyed by Snx4-assisted autophagy following TORC1 inhibition.	[42]	unknown

## Data Availability

Not applicable.

## Abbreviations

Å	Angstrom
AGC-kinase	group of kinases related to PKA PKG and PKC
AMPK	adenosine monophosphate-activated protein kinase
ATP	adenosine triphosphate
CAK	CDK-activating kinase
CC	cyclin C
CDK	Cyclin-dependent kinase
CDKI	cyclin-dependent protein inhibitor
Cryo-EM	Cryogenic electron microscopy
EGOC	Gtr1, Gtr2, Ego1, and Ego3 complex that can activate TORC1
ERK5	extracellular signal-regulated kinase 5
GTP	guanosine triphosphate
HDAC	Rpd3–Sin3–Ume6 histone deacetylase
HOG	high osmolarity glycerol
IDR	intrinsic disordered region
IMM	inner mitochondrial membrane
MAPK	mitogen-activated protein kinase
MAPK	mitogen-activated protein kinase kinase
MAPKKK	mitogen-activated protein kinase kinase kinase
MEFs	mouse embryonic fibroblasts
MFF	mitochondrial fission factor
MOMP	mitochondrial outer membrane permeabilization
mtDNA	mitochondrial DNA
NPC	nuclear pore complex
OMM	outer mitochondrial membrane
OXPHOS	oxidative phosphorylation
PAS	pre-autophagosomal structure
PIC	pre-initiation complex
Pkc1	protein kinase C
PKA	protein kinase A
RNA Pol II	RNA Polymerase II
ROS	reactive oxygen species
SAA	Snx4-assisted autophagy
SCF	Skp1-Cullin-F-box E3 ligase complex
SRG’s	stress response genes
SEACIT	Sea1–Npr2–Npr3 complex that can inhibit TORC1
Ser	serine
TORC1	target of rapamycin kinase complex 1
Thr	threonine
TF	Transcription factor
UAS	upstream activating sequences
UPS	ubiquitin proteasome system
YFP	yellow fluorescence protein
